# The Future of the Weight‐of‐Evidence Approach: A Response to Suter's Comments

**DOI:** 10.1002/etc.5215

**Published:** 2021-10-26

**Authors:** Andrew C. Johnson, John P. Sumpter, Michael H. Depledge

**Affiliations:** ^1^ UK Centre for Ecology and Hydrology Wallingford United Kingdom; ^2^ Institute of Environment, Health and Societies, College of Health and Life Sciences Brunel University London Uxbridge United Kingdom; ^3^ European Centre for Environment and Human Health University of Exeter Medical School, Knowledge Spa, Royal Cornwall Hospital, Truro Cornwall United Kingdom

Authors’ Response:

We are grateful to Suter (2021) for noting our effort (Johnson et al., 2021) and for the role that he and his co‐workers have played (and continue to play) in championing the weight‐of‐evidence (WoE) approach. As Suter has pointed out, the field is far from stagnant and enjoys continuing debate within parts of the community (Suter, 2021). With regard to his response to our article, we see no reason to criticize the Hill criteria (Hill, 1965) but find that, while agreeing with Suter that they are not a perfect fit for our field, they are of enduring value and are readily adaptable.

We consider that there remain a number of issues that both hinder the take‐up of WoE and have yet to be resolved in the decision‐making process over whether a chemical is a threat or not. We suggest reasons behind the worldwide poor take‐up of WoE.

## SKILL SETS AND MIND SETS

The majority of scientists working in the field of chemicals in the environment are laboratory scientists with limited knowledge of field studies, long‐term monitoring, or ecology. Therefore, the instincts and skills needed to understand field evidence (an essential component of WoE) are not widespread. Many scientists refer to and rely on their own data coming out of their laboratories. Reviewing long‐term monitoring data collected by third parties may be mistrusted. The fields of genomics, proteomics, and metabolomics offer extraordinary new windows into how the biological machine works. This has led many to place confidence into “adverse outcome pathways” as a reliable guide to field effects. We do not share this confidence. The appeal of funding new technologies as the way to make progress in the study of chemicals in the environment can outshine requests for long‐term monitoring support which, in some cases, use methods that have been largely unchanged for over 100 years. Also, molecular approaches often neglect elements such as chemically induced behavioral changes which can influence growth, reproduction, and survival of offspring, resulting in profound ecological change without overt toxicity. Despite its cadre of enthusiasts, we perceive that the intellectual drive and discussions on WoE remain narrow. This has knock‐on effects in education and reduces the likelihood that WoE as a methodology will be taught in undergraduate, master's, or PhD courses. The lack of take‐up and understanding of the WoE approach are thus perpetuated.

## FUNDING MODELS

Research funding organizations, if not government agencies, examine chemicals in the environment mainly through 3‐year research grants which tend to be laboratory studies where key variables are controlled. Thus, the complexities of how animals respond in the field are rarely researched. Research funding organizations do not tend to want to tie their money up in longer‐term monitoring schemes so important to WoE. Similarly, government agencies are constantly under pressure to cut costs and avoid long‐term commitments so that national chemical and wildlife monitoring efforts are themselves in decline (as is evident in the United Kingdom). We appeal to all those both within the field of chemicals in the environment and who have influence over its future to review how we gather evidence and consider whether we have got the funding emphasis and strategy right.

## HOW BIAS CAN UNDERMINE EFFORTS TO REACH BALANCED CONCLUSIONS

Of the many voices that input into WoE, scientists are usually regarded as the most rational, objective people, able to think critically and reach informed, robust conclusions (Johnson & Sumpter, 2019). Yet the existence of disagreements on many major issues in (eco)toxicology demonstrates that scientists often interpret data subjectively, sometimes in profoundly different ways. For example, ecotoxicologists do not agree on whether or not a low concentration of a chemical can cause an adverse effect that does not occur when exposure is to higher concentrations; they appear to be split about 50:50 on this key issue (Tanoue et al., 2019). These disagreements, which sometimes lead to very robust debates (e.g., the arguments over atrazine and its possible adverse effects on amphibians), illustrate what psychologists term confirmation bias, which is the tendency to search for and then interpret and favor information in a manner that confirms one's prior opinions. It is not deliberate deception; it is an example of cognitive bias. Beliefs—such as “I know that chemical A causes effect B”—can be very deeply entrenched, and hence very difficult to relinquish. The literature can reflect a reluctance of scientists and academic journals to publish papers reporting few or no effects of a chemical; thus, the message that it actually presents low or no risk to exposed organisms can get overlooked (Hanson et al., 2018). Similarly, the claim that there is “no evidence” of adverse effects can sometimes literally mean there really is no evidence because nobody has conducted a study. This is an equally dangerous misinterpretation relevant to real‐world situations. Skewing of the literature can unwittingly strengthen the bias of some scientists that chemical A is causing effect B (or that it is not, depending on the circumstances), and thus imperil some populations. If the WoE approach is to become more widely used and generally accepted, it will be necessary for those conducting the assessments to be aware of biases and approach their task with dispassion.

## REVIEWING THE PERFORMANCE OF THE WoE APPROACH

With our eyes firmly set on substantially improving chemical management over the coming years, it is important that we review the performance of the WoE approach in a range of real‐world situations. Checking whether the “right” decisions were made when WoE was used is crucial. Confirmation can only be achieved by significantly increasing environmental and human biomonitoring to gather reliable evidence that biodiversity and ecosystem structure and function have actually been protected from chemical toxicity over the long term and that no unanticipated human health effects ensued. This should provide more confidence in the robustness of an approach that integrates environment and human health risk assessment procedures (Reis et al., 2015). All assessments, like those purporting to be via WoE, should not be accepted at face value without independent scrutiny. To address these and other concerns, we agree with Suter et al. (2020) that systematic review is a potentially valuable tool for checking the reliability of studies, but we caution that in medical sciences, where this procedure is used extensively, the reviews themselves are often poorly designed and lacking in quality (Ioannidis, 2016). When deficient, they provide an unsuitable basis for decision‐making.

## SPREADING THE TASK OF WHO JUDGES CHEMICAL RISKS AND THE NEED FOR PROTECTION?

Governments entrust chemical risk assessments to regulators. To ensure consistency, they will follow strict guidelines. Such reviews tend to exclude many external stakeholders or experts from participation. It would be helpful to society as a whole if the approaches taken by regulators or policymakers to assess risk were to be routinely scrutinized, debated, and, where necessary, refreshed by the widest possible range of stakeholders and experts. Biases present in one group of representatives (e.g., scientists from industry or academic scientists with no fieldwork experience) would then be balanced against a different set of biases present in another group of representatives. There would also be greater acceptance of the conclusions because all stakeholders would have participated in the process; none would feel excluded. A final advantage could be that the process of conducting WoE assessments could be speeded up because the work would be shared by a wider group of experts.

## ENGAGING THE PUBLIC AS WE CONSIDER THE FUTURE OF CHEMICALS IN THE ENVIRONMENT

Retrospective and prospective risk assessments are becoming increasingly important as global chemical production and release into the environment continue to rise exponentially (Collins et al., 2020). The WoE approach allows us to identify and hopefully remedy damage from stressors like chemicals by bringing together the widest range of scientific data available. We are less adept at revealing the economic, legal, and social implications of a chemical's use, particularly over the long term (decades). The challenge in the years ahead is to ensure that through a step change in engagement, the public, policymakers, and politicians are sufficiently well informed about these matters to be able to contribute and sensibly decide what is and what is not acceptable in terms of threats posed by environmental chemicals. Presenting the WoE in a way that informs opinion and but also tests society's willingness to be exposed to low concentrations of mixtures of environmental chemicals, potentially across the entire life course of wildlife and humans, is a challenge we have yet to fully address.

## FINAL THOUGHTS

There remain many issues where groups of wildlife are in decline, such as eels, migratory salmonids, seabirds, marine mussels and barnacles, terrestrial insects, and farmland birds in which chemicals are thought to play a contributory role. Yet in these and in other cases we have considerable amounts of monitoring data and information on potentially important variables collected over decades. Perhaps if we were to apply WoE approaches with greater confidence to these and related topics, we might make more progress.
